# Wild Honey Intoxication: A Case Series From Nepal

**DOI:** 10.1002/ccr3.70628

**Published:** 2025-07-11

**Authors:** Rukma Raj Kafle, Rakshya Arun Kandel, Sabin Chaulagain, Angela Basnet, Shiva Kumar Ojha, Alok Atreya

**Affiliations:** ^1^ Department of Emergency Medicine Scheer Memorial Adventist Hospital Banepa Nepal; ^2^ Department of Internal Medicine Scheer Memorial Adventist Hospital Banepa Nepal; ^3^ Department of Forensic Medicine Lumbini Medical College Palpa Nepal

**Keywords:** bradycardia, grayanotoxins, honey, hypotension, Nepal, rhododendron

## Abstract

Wild honey poisoning can cause serious cardiovascular complications, including bradycardia and hypotension, even with small amounts (1–2 teaspoons) consumed. Symptoms typically emerge within 30–45 min of ingestion. Early intervention with IV fluids and atropine is effective in most cases, but severe cases may require vasopressors, particularly when presentation is delayed.

## 1 Introduction

Wild (mad) honey poisoning is caused by the ingestion of honey containing toxins known as grayanotoxins, which is derived from the nectar of various *Rhododendron* species found mainly in higher altitude regions of Nepal, India, and Turkey [1]. The Himalayan cliff honey bee scientifically known as *Apis dorsata laboriosa* is the largest honey bee in the world which is responsible for mad honey production in Nepal's Himalayan region [2]. The Royal Botanic Gardens in London reported that the world is home to a total of 173 species of *Rhododendron*. Among them, 33 are found in Nepal across 43 districts in hilly and mountainous regions [3]. Out of these 33 species, four particular species, *R. luteum* , *R. ponticum*, *R. simsii*, and *R. flavum*, contain varying levels of grayanotoxins [4]. These toxins are then transferred to honey when the bees collect nectar from these rhododendrons. People consume it for various cosmetic and medical purposes, especially for gastrointestinal disorders (peptic ulcer disease, dyspepsia, and gastritis), hypertension, aphrodisiac (sexual stimulant), diabetes, flu, viral infections, colds, and arthritis [5]. Mad honey, which is found in Nepal, consists mostly of the grayanotoxin I and III isoforms, which interact with the voltage‐gated sodium channels by binding the channels in the activated state, leading to prolonged depolarization and hyperpolarization effects [6]. Mad honey poisoning is diagnosed clinically and can cause minor side effects, such as nausea, vomiting, dizziness, blurred vision, diplopia, sweating or excessive perspiration, headache, impaired consciousness, extremity paresthesia, convulsions, hypersalivation, ataxia, inability to stand, and general weakness to major cardiac side effects, such as sinus bradycardia, hypotension, nodal rhythms, and various degrees of atrioventricular block [6]. While the precise amount of wild honey needed to induce toxicity remains undefined, clinical observations suggest that consuming as little as one teaspoonful can trigger symptoms [7]. Recent reports have highlighted additional complexities, such as the exacerbation of symptoms when mad honey is consumed with alcohol, leading to severe presentations including syncope and pulmonary abnormalities [8]. Owing to its ability to combat various diseases, its consumption has increased readily in the past few years, leading to an increase in cases of mad honey poisoning. Here, we present two cases of mad honey poisoning within one month, highlighting clinical variability and management strategies.

## 2 Case Presentation

### 2.1 Patient 1

History: A 65‐year‐old male presented to the department of emergency (ED) with complaints of nausea, two episodes of vomiting, bilateral upper and lower limb weakness, diplopia, intermittent blackouts, chest discomfort, and burning abdominal pain for one day after consuming two teaspoons full (30 mL) of wild honey in the morning and evening the previous day. The symptoms started 30–45 min after ingesting the first dose, but due to transportation issues, the patient reached the hospital 26 h after the initiation of symptoms. There was no history of trauma or use of alcohol or drugs. The patient's medical history was significant for essential hypertension, managed with daily oral losartan 25 mg for the past six months.

**FIGURE 1:**
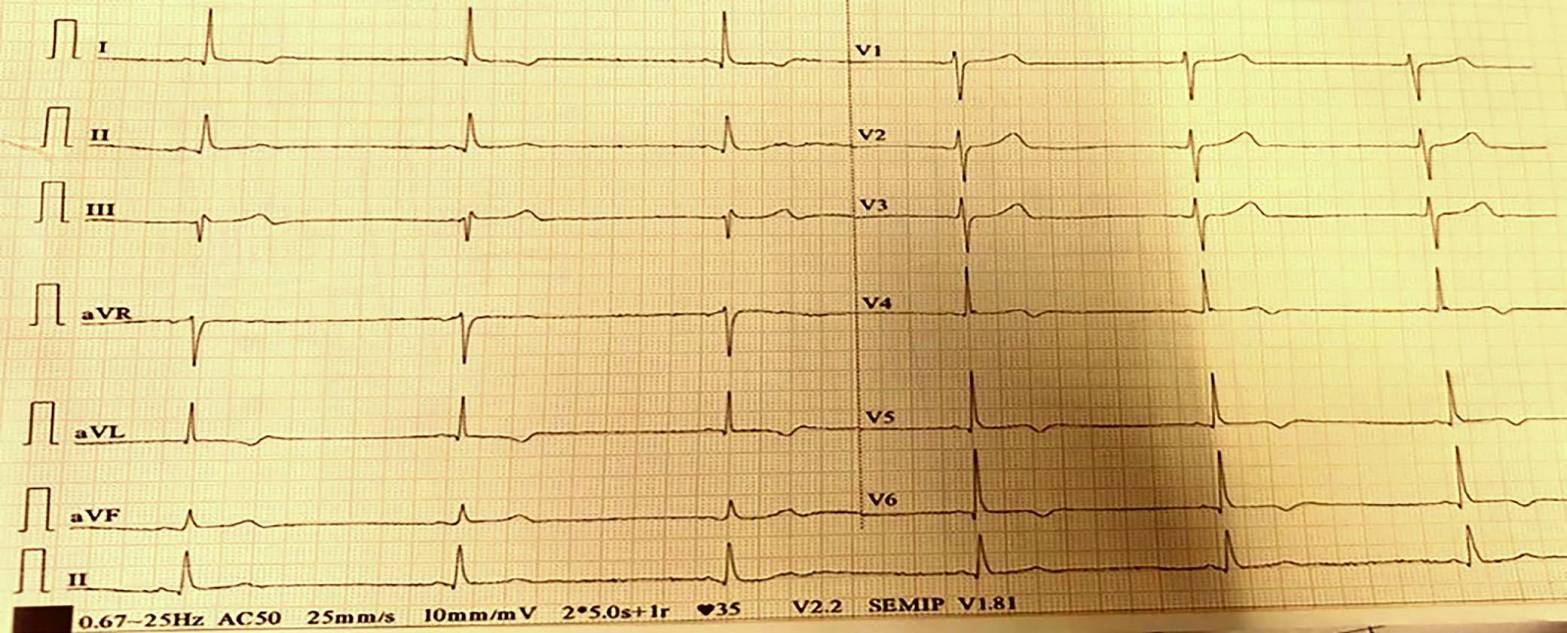
Electrocardiogram of Patient 1 at presentation, showing sinus bradycardia, consistent with grayanotoxin‐induced vagal overstimulation.

Examination: Initial assessment revealed the patient was conscious, and his pupils were bilaterally dilated and reactive to light. His blood pressure (BP) was 60/40 mm of Hg, his pulse rate was 35 beats per minute (bpm), his temperature was 98.7°F (37.05°C), his respiratory rate was 16 breaths per minute, and his oxygen saturation was 97% at room air. Neurological examination revealed that the patient had mild confusion (13/15 on the Glasgow coma scale). Other systemic examinations were within the normal limits. A 12‐lead electrocardiogram (EKG) revealed sinus bradycardia with a heart rate of 41 bpm without any conduction delays (Figure 1). Differential diagnoses, including acute coronary syndrome, other toxic ingestions, and electrolyte imbalances, were ruled out based on history, normal laboratory results, and EKG findings.

### 2.2 Patient 2

History: A 35‐year‐old male presented to ED with complaints of dizziness and an episode of syncopal attack 20 min prior to presentation. A further history revealed that symptoms appeared 20–25 min after the ingestion of two tablespoons full (25–30 mL) of wild honey mixed with warm water. He had consumed it to get relief from the common cold from which he was suffering for a day. There was no history of abdominal pain, nausea, vomiting, diplopia, sweating, excessive perspiration, or headache. There was no history of trauma or use of alcohol or drugs. The patient did not have any comorbidities and was not on any regular medications.

**FIGURE 2:**
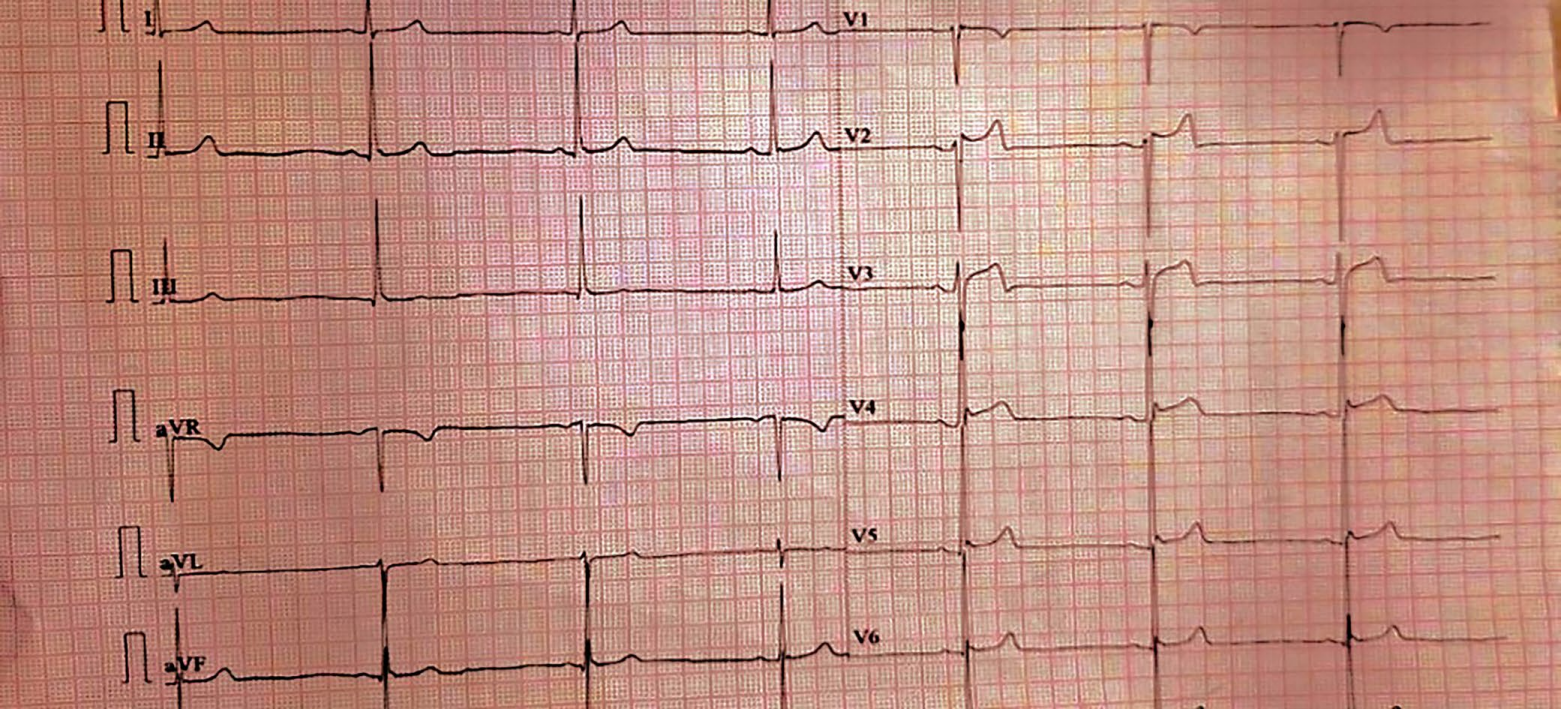
Electrocardiogram of Patient 2 at presentation, demonstrating sinus bradycardia, reflecting the cardiovascular effects of mad honey intoxication.

Examination: On examinations, he was conscious and well oriented to person, place and time. His BP was 80/50 mm of Hg, his heart rate was 44 bpm, and his respiratory rate was 20 breaths per minute. The patient was afebrile and had an oxygen saturation of 98% in room air. EKG performed immediately revealed sinus bradycardia with a heart rate of 41 bpm (Figure 2). Other causes of bradycardia and hypotension, such as beta‐blocker overdose or hypovolemia, were excluded based on history and clinical findings.

## 3 Methods

### 3.1 Diagnostic Approach

- Clinical diagnosis based on the history of honey consumption and characteristic symptoms (bradycardia, hypotension, and neurologic complaints);
- No specific laboratory test confirms grayanotoxin poisoning, so other causes of bradycardia and hypotension (e.g., myocardial infraction, hypovolemia, sepsis, beta‐blocker overdose, or organophosphate poisoning) were ruled out through clinical history, physical examination, and laboratory investigations;
- 12‐lead EKG revealed sinus bradycardia in both patients, supporting the diagnosis.

### 3.2 Patient 1 Treatment

Within 10 min of arrival, resuscitation began with 1 L of intravenous (IV) normal saline (0.9%), IV atropine (1 mg stat), and IV hydrocortisone (100 mg stat). Laboratory results were normal. Upon transfer to the intensive care unit (ICU), continuous monitoring was initiated alongside fluid resuscitation, scheduled hydrocortisone (100 mg every 8 h), and as‐needed atropine for severe bradycardia (threshold: heart rate below 40 bpm). Despite aggressive volume expansion with 3 L of crystalloid solution (1500 mL in the ED and 1500 mL in the ICU), the patient remained hypotensive. His systolic readings fluctuated between 60 and 80 mm of Hg, diastolic pressure ranged from 50 to 60 mm of Hg, and heart rate persisted between 50 and 60 bpm. Noradrenaline (6 cc/h) was initiated 4 h after admission, stabilizing BP to 110/70 mmHg and heart rate to 70–88 bpm within 6 h.

### 3.3 Patient 2 Treatment

The patient received 1 L of IV normal saline (0.9%) and IV atropine (1 mg stat) within 10 min of arrival. No further atropine or vasopressors were needed, as vitals improved rapidly.

## 4 Results

### 4.1 Patient 1 Outcome

Within 24 h of intensive care management, the patient achieved hemodynamic stability (BP 120/80 mmHg, pulse 74 bpm) and was successfully weaned off vasopressor support. The patient was transferred to the general ward on Day 2 and discharged on Day 4 with stable vitals after 72 h of observation to ensure symptom resolution.

### 4.2 Patient 2 Outcome

Following treatment, his hemodynamic parameters stabilized with BP reaching 100/60 mmHg and heart rate increasing to 94 beats per minute. The patient was monitored in ICU for 24 h, then transferred to the general medical ward for an additional 24 h. He was discharged on Day 3 with normal vitals after 72 h of observation.

Both patients achieved hemodynamic stability within 24 h, with Patient 1 requiring vasopressor support due to delayed presentation and Patient 2 recovering with simpler interventions. A summary of diagnostic and management points for both cases is presented in Table 1.

**TABLE 1:** Summary of diagnostic and management points for mad honey poisoning cases.

Parameter	Patient 1	Patient 2
Age/sex	65/male	35/male
Honey amount	30 mL (2 teaspoons)	25–30 mL (2 tablespoons)
Symptoms	Nausea, vomiting, limb weakness, diplopia, blackouts, chest discomfort, and abdominal pain	Dizziness, syncope
Time to symptom onset	30–45 min	20–25 min
Initial BP (mmHg)	60/40	80/50
Initial heart rate (bpm)	35	44
EKG findings	Sinus bradycardia (41 bpm)	Sinus bradycardia (41 bpm)
Treatment	IV saline, atropine (1 mg), hydrocortisone, and noradrenaline	IV saline, atropine (1 mg)
Escalation criteria	Noradrenaline for refractory hypotension (BP < 80/60 mmHg after 3 L fluids)	Not required
Outcome	Stabilized in 24 h, discharged after 72 h	Stabilized in 24 h, discharged after 48 h

## 5 Discussion

Honey, a natural product of bees processing plant nectar, has been used since 2100 bc for conditions like diabetes, hypertension, common colds, arthritis, sexual stimulation, ulcers, wound healing, and gastritis [9]. In some cases, it has also been used for recreational purposes. Toxic compounds present in certain types of honey can result in adverse reactions when consumed. Two distinct forms of honey toxicity have been documented: the first occurs from ingesting honey contaminated with grayanotoxins, which are produced by *Rhododendron* plants, leading to what is commonly known as “mad honey poisoning”—predominantly seen in Nepal, Turkey, and Korea [5]. The second type stems from honey containing the neurotoxin compound tutin, derived from *Coraria* shrubs, with cases primarily documented in New Zealand [10]. In Nepal, Rhododendron, the national flower is responsible for the toxic effect of wild honey due to grayanotoxins, which are considered the most potent forms of toxins [11, 12, 13]. Literatures suggest that more than 20 types of gryanotoxins are identified [14]. Grayanotoxins interact with voltage‐gated sodium channels in excitable cells, particularly in the heart and nervous system [15]. By binding to these channels in their open state, graynotoxins prevent inactivation that results in sustained membrane depolarization [15]. In the autonomic nervous system, this enhances parasympathetic activity, increasing vagal tone, and subsequent cardiovascular effects including sinus bradycardia and hypotension [15]. While most individuals exhibit parasympathetic nervous system effects, a subset may develop severe cardiac manifestations [16]. A review of nearly 1200 cases showed hypotension as the primary clinical manifestation, affecting approximately 20% of patients, while electrocardiographic evaluation showed predominant sinus bradycardia, observed in about 80% of patients [17]. A case series reports seven patients; most patients complained of blurring of vision, with only two cases had symptoms of cardiac depression [18]. Although the exact amount of honey required for poisoning is not yet known, symptoms have been reported to occur 0.5–3 h post‐ingestion even with a small dose (1 tablespoon) [19]. Notably, the clinical presentation may be exacerbated by additional factors, such as the co‐ingestion of alcohol. In another case, a middle aged women developed visual hallucinations in addition to cardiovascular and neurological symptoms [20]. A case is reported from Nepal where the combination of aged honey and alcohol resulted in profound hypotension, bradycardia, and bilateral lung haziness, suggesting broader systemic effects beyond typical cardiovascular manifestations [8]. This finding highlights the potential for atypical presentations, including pulmonary involvement, which was not observed in our cases. In both of our cases, the patients consumed more than one teaspoon full of mad honey, and in both cases, the symptoms started after 30 min of consumption. However, the severity depended upon the time of arrival at the hospital and the cumulative dose. As in Patient 1, the patient also took a second dose of mad honey in the evening and was brought to the hospital after 26 h of ingestion of the first dose.

The diagnosis of mad honey intoxication is performed clinically on the basis of a history of ingestion and associated symptoms. It is suspected that patients who have no record of heart disease earlier present with bradycardia, hypotension, nausea, vomiting, and syncope after consumption of honey [21]. Symptomatic treatment is adopted in most cases of mad honey intoxication, and for severe hypotension and bradycardia, atropine is preferred. Treatment of mad honey poisoning typically involves IV saline for hypotension and atropine for bradycardia, with vasopressors like dopamine used in severe cases, as observed in our patients and supported by prior reports [14, 22]. In cases where patients remain unresponsive to initial treatment with saline and atropine, escalation to vasopressor therapy (e.g., adrenaline, noradrenaline, or dopamine) or temporary transvenous cardiac pacing may be necessary [23]. Both patients received a single dose of 1 mg atropine in the ED, and Patient 1 also received a noradrenaline infusion for refractory hypotension.

In most cases, the symptoms last for only 24 h [24]. However, in one of the patients, the symptoms persisted for up to 72 h and had to be readmitted and monitored in the ICU [15]. Based on these experiences and previous reports of extended symptom duration lasting up to 72 h, we maintained careful observation of both patients for a full 72‐h period to ensure complete resolution of symptoms.

Wild honey hunting has emerged as a ludicrous business in Nepal because the wild honey has a good market in Japan and Korea, where it is consumed for its medicinal properties [25]. Furthermore, the challenging act of honey hunting in the wild is considered an adventurous sport by foreign tourists in the country [26]. The honey thus collected is a prized reward and consumed for the feeling of accomplishment and intoxicating effect [26].

Public health initiatives should focus on community education programs in Nepal's mountainous regions, using workshops and local media to highlight the risks of wild honey consumption and promote early medical consultation for symptoms.

## 6 Conclusions

This case series shows that mad honey poisoning in Nepal can have a spectrum of predictably mild symptoms, such as dizziness and syncope, to more severe manifestations that may require vasopressor support. In both cases, symptom onset typically occurs within 30–45 min of consumption, even with relatively small amounts (2 teaspoons) of wild honey. Although Patient 2 required only basic medical intervention (IV fluids and atropine) and recovered within 24 h, the more serious clinical condition of Patient 1 requiring vasopressors reiterates the need for early and specific medical intervention but may indicate that a delay in initial presentation (26 h with this patient) leads to greater severity. Healthcare providers in the mountainous regions of Nepal should suspect mad honey poisoning in unexplained bradycardia and hypotension, especially given its medicinal use. The cases further highlight the need for public health awareness on the potential risks posed by wild honey consumption and the importance of early clinical attention when symptoms arise.

## Author Contributions

**Rukma Raj Kafle:** data curation, writing – original draft. **Rakshya Arun Kandel:** conceptualization, data curation, writing – original draft, writing – review and editing. **Sabin Chaulagain:** data curation, writing – review and editing. **Angela Basnet:** data curation, writing – review and editing. **Shiva Kumar Ojha:** data curation, writing – review and editing. **Alok Atreya:** writing – original draft, writing – review and editing.

## Ethics Statement

Institutional policy waives ethics committee approval for case reports. Patient confidentiality was maintained by de‐identifying all personal information and including only the essential clinical details, such as age and medical history.

## Consent

Written informed consent was obtained from the patients for the use of images and medical history for educational purposes, including publication.

## Conflicts of Interest

The authors declare no conflicts of interest.

## Data Availability

All data underlying the results are available as part of the article, and no additional source data are required.
